# Effectiveness of Platelet-Rich Plasma Injection in Rotator Cuff Tendinopathy: A Systematic Review and Meta-Analysis of Randomized Controlled Trials

**DOI:** 10.3390/diagnostics10040189

**Published:** 2020-03-28

**Authors:** Meng-Ting Lin, Kuo-Chang Wei, Chueh-Hung Wu

**Affiliations:** 1 Department of Physical Medicine and Rehabilitation, National Taiwan University Hospital, College of Medicine, National Taiwan University, No.7, Zhongshan S. Rd., Zhongzheng Dist., Taipei 100, Taiwan; 2 Department of General Medicine, National Taiwan University Hospital Biomedical Park Branch, Hsinchu 300, Taiwan

**Keywords:** platelet-rich plasma, injections, rotator cuff, tendinopathy

## Abstract

Background: To investigate the effectiveness of platelet-rich plasma (PRP) injection among patients with rotator cuff tendinopathy in comparison with sham injection, no injection, or physiotherapy alone. Methods: From the earliest records to November 1, 2018, all published or unpublished randomized controlled trial (RCTs) comparing PRP injection with a control group (sham injection, no injection, or physiotherapy alone) in patients with rotator cuff tendinopathy were included. Eligible trials were included from the search results of electronic databases including PubMed, EMBASE, Scopus, The Cochrane Library, as well as the bibliographies of relevant trials. Five RCTs were enrolled in our meta-analysis. Two authors independently assessed the quality of RCTs with the Cochrane risk of bias tool. We designated pain reduction as the primary outcome and functional improvement as the secondary outcome. Standardized mean difference (SMD) was applied for random-effect meta-analysis. Results: In the short term (3–6 weeks) and medium term (12 weeks), the effectiveness of PRP injection and control group was indistinguishable in terms of both outcomes (pain reduction and functional improvement). Nevertheless, PRP injection led to significant long-term (>24 weeks) pain relief (SMD: 0.42, 95% confidence interval (CI): 0.12–0.72, without heterogeneity). For functional improvement in the long term, PRP injection was not more effective than the control group (SMD: 1.20, 95% CI: −0.20–2.59, with heterogeneity). Conclusions: PRP injection may provide benefit over the control group (sham injection, no injection, or physiotherapy alone) in reducing pain at long-term follow-up for patients with rotator cuff tendinopathy.

## 1. Introduction

Rotator cuff tendinopathy, characterized by its chronic degenerative process, causes the shoulder pain and deterioration of daily activities [1]. The effectiveness of conservative treatments such as physiotherapies (exercise and manual therapy) has been widely established in pain reduction and functional improvement in the past few years [2,3,4,5]. In addition to exercise therapy, various injection options including corticosteroid and non-corticosteroid injections are available in pain reductions [6].

Platelet-rich plasma (PRP) is obtained by the centrifugation of whole blood into its component fractions, which has a higher concentration of platelets above that of the baseline [7]. PRP contains specific growth factors that exist in the blood to promote healing. The growth factors integral in the healing process include transforming growth factor b1, platelet-derived growth factor, vascular endothelial growth factor, hepatocyte growth factor, and insulin-like growth factor 1 [7]. PRP has been shown to provide beneficial effects on musculoskeletal diseases including chronic joint degeneration and tendinopathy [8,9]. Some new emerging randomized controlled trials (RCTs) regarding PRP injection for rotator cuff tendinopathy have been published recently [10,11,12,13,14]. In addition, positive evidence supporting the efficacy of PRP for treating rotator cuff tendinopathy has been reported in a previous review, but no comparison with conventional therapies such as physiotherapy was made [15]. Still physiotherapy was considered as one of standard treatments in rotator cuff tendinopathy with improvement in functional outcome being sustained for 6 months [16]. To our knowledge, this is the first meta-analysis with physical therapy considered as the control group.

In this meta-analysis, we aim to investigate the effectiveness of PRP injection regarding pain reduction and functional improvement compared with the control group including sham injection, no injection, or physiotherapy in adult patients with rotator cuff tendinopathy.

## 2. Methods

The systematic review and meta-analysis of this study were conducted in accordance with the recommendations from the Preferred Reporting Items for Systematic Reviews and Meta-Analyses for Network Meta-Analyses (PRISMA-NMA) [17]. The review protocol of this meta-analysis was registered at PROSPERO (http://www.crd.york.ac.uk/prospero/).

### 2.1. Study Identification and Search Method

Eligible trials were included from the search results of electronic databases including PubMed, EMBASE, Scopus, The Cochrane Central Register of Controlled Clinical Trials, the Cochrane Database of Systematic Reviews, as well as the bibliographies of relevant trials. Our search was from the earliest records to 1 November 2018. In addition, further references of relevant systematic reviews were manually searched. Relevant grey literature was searched using clinicalTrials.gov, OpenSIGLE (www.opengrey.eu), and the New York Academy of Medicine Grey Literature Report (www.greylit.org). Our search was not limited to articles published in the English language. The search strategies are listed in detail in the Supplementary Methods.

### 2.2. Eligibility Criteria

#### 2.2.1. Types of Studies

All published or unpublished RCTs were included. RCTs that were quasi-experimental trials, observational studies, case series, single-arm trials, or animal studies were excluded.

#### 2.2.2. Participants

We included studies in which adult participants were diagnosed with rotator cuff tendinopathy by clinical or image evaluation. In accordance with previous systematic reviews, the disease entity of rotator cuff tendinopathy comprised tendinosis, partial tear of rotator cuff, and impingement syndrome [6,18]. Studies that included subjects of adhesive capsulitis, trauma, full-thickness tears, calcific rotator cuff disease, or rheumatological disease were excluded [6]. 

#### 2.2.3. Interventions

RCTs that had allocated a PRP-treated group and a control group were eligible for inclusion. The control group included sham injection, no injection, or physiotherapy alone. The number of injections or the injection-guiding technique was not limited.

#### 2.2.4. Outcomes

We designated pain reduction as the primary outcome and functional improvement as the secondary outcome. All validated measures of shoulder function and pain were feasible. The post-interventional follow-ups were assigned into three groups: 3–6 weeks (short term), 12 weeks (medium term), and over 24 weeks (long term).

#### 2.2.5. Data Extractions

M.-T.L. and K.-C.W. independently reviewed the eligibility for the inclusion of all related studies. Inter-rater reliability was evaluated by the kappa statistic. Disagreements were resolved through consensus-based discussion among all three authors. Information including the number of patients, age, symptom duration, injection interval, dosage, guidance technique, injection location, co-interventions, follow-up, and adverse effects was assessed from the included studies. Data with means, standard deviations (SD), and the number of participants were extracted for outcome measurements. If the data were not accessible, not extractable, or were expressed in formats other than mean and SD, the corresponding author was contacted through e-mail every 3 weeks for up to three times.

### 2.3. Risk of Bias Assessment

The quality of RCTs was evaluated using the Cochrane risk of bias tool, as described in the Cochrane Handbook for Systematic Reviews of Interventions [19]. Possible biases are divided into five domains (selection bias, performance bias, detection bias, attrition bias, and reporting bias) and a generalized category of other biases. All items were assessed by two authors independently. Each outcome within a study across domains and each outcome across the studies was rated as having “Low risk”, “Unclear risk”, or “High risk” of bias [19]. Inter-rater reliability was determined by the kappa statistic to evaluate the strength for the risk of bias assessments. Discussions with the corresponding author were made to solve the disputes. 

### 2.4. Data Synthesis and Analysis

The effectiveness of PRP injection was calculated as the difference between baseline measurement and post-injection outcome: Measurement baseline—Measurement post-injection. The standardized mean difference (SMD) was adopted for proper comparison of different outcome scales or questionnaire measurements. Formulation of pooled SD between-injections is described in the Supplementary Methods. A positive value of the SMD indicated that PRP injection was more effective than control treatment [19]. A random effect model was used to calculate the pooled SMD with a 95% confidence interval (95% CI). The heterogeneity was assessed by I-square and Cochran’s Q tests. An I-square (I^2^) over 50% was recognized as significant heterogeneity [19]. Publication bias, defined as the tendency for trials with positive results to be published and for negative and null trials to be unpublished, was evaluated by a funnel plot [20]. A sensitivity analysis was executed by excluding low-quality studies. We performed the meta-regression to examine the relation between number of injections and outcomes improvement (pain reduction and functional recovery). The analysis was performed using Stata 14.0 (StataCorp LP, College Station, TX, USA) and Review Manager 5.3 (RevMan, Cochrane Collaboration, Copenhagen, Denmark). All *p* values were two-sided, and the significance level was set at 5% except for the test of between-study heterogeneity.

## 3. Results

### 3.1. Characteristics of the Included Studies

**Figure 1:**
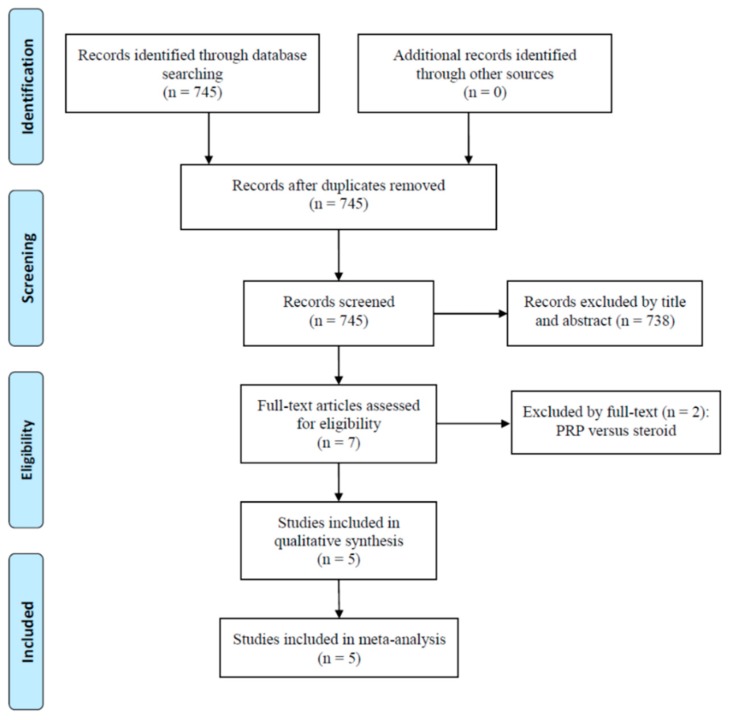
A flow diagram of study inclusions. PRP, platelet-rich plasma.

Initially, 745 studies were identified from electronic databases. No duplicates were found and all 745 studies were screened. Seven full-text articles were evaluated for eligibility (Figure 1). We excluded two studies comparing PRP to corticosteroid [21,22]. Five RCTs were included for the qualitative synthesis as well as the final meta-analysis (Table 1) [10,11,12,13,14]. The comparison between the PRP and control group in this study comprised 283 patients [10,11,12,13,14]. Two of the included studies compared PRP with physiotherapy [12,13], another two studies compared PRP with saline injection [11,14], and one study investigated the comparison between PRP and dry needling [10]. 

The mean age of the enrolled patients ranged from 39.9 to 59.7 years in the five included studies. The information regarding symptom duration was not available in one study [13], and the symptom duration varied from 13.5 weeks to 10 months in the other studies. Rotator cuff lesions were diagnosed with ultrasonography in one study [10] and were diagnosed clinically or via magnetic resonance imaging (MRI) in the other four studies [11,12,13,14]. The outcomes were extracted at baseline and different follow-up time points for all studies. Among the five studies, four used ultrasound for injection guidance [10,11,13,14]. Regarding the injection site, two RCTs performed subacromial injections [11,14], one with supraspinatus injections [10], one with injured tendon and subacromial injections [13], and one with intra-articular injections [12]. For four of the included RCTs, repeated PRP injections from 2–4 times were given with intervals ranging from 1 week to 1 month [10,12,13,14], while PRP was only administered once for each patient in the remaining study [11]. 

### 3.2. Risk of Bias Assessment

**Figure 2:**
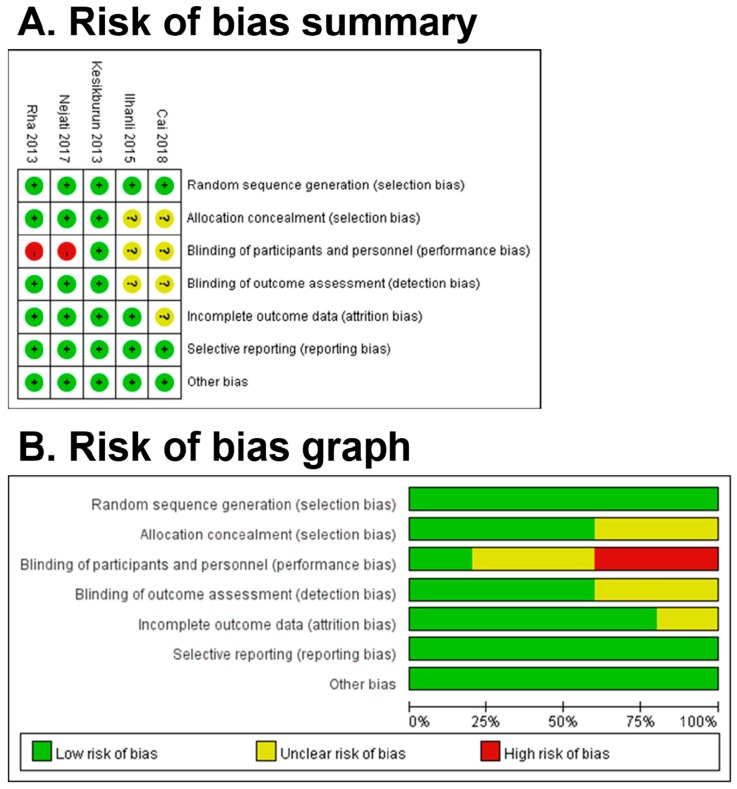
Risk of bias summary (**A**) and graph (**B**). Green, low risk of bias; red, high risk of bias; yellow, unclear risk of bias.

The graph and summary of the risks of bias are presented in Figure 2. Most of the high or unclear risks of bias were generated in the blinding of participants and personnel. It was difficult to blind the patients due to blood drawing and PRP preparation. In allocation concealment, two studies yielded unclear risk of bias because there was no description provided for the method of concealment. Regarding the detection bias, two articles did not depict whether the outcome assessors were blinded to treatment groups or not.

### 3.3. Results of Meta-Analysis: Primary Outcome (Pain Reduction) 

**Figure 3:**
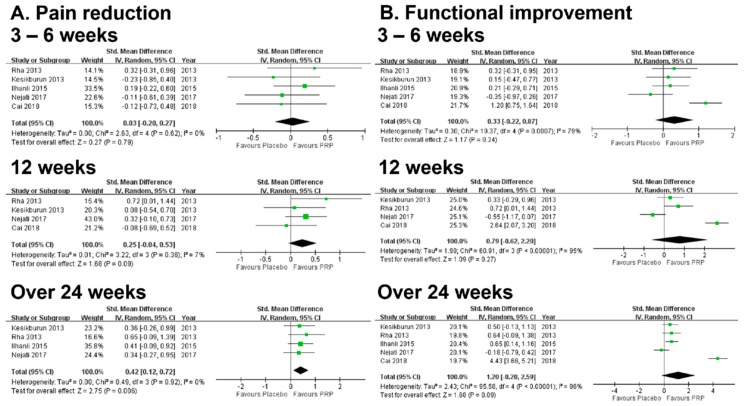
Forest plots of meta-analysis after sensitivity analysis: comparison between PRP injection and the control group in pain reduction (**A**) and functional improvement (**B**) at the short term (3–6 weeks), the medium term (12 weeks), and the long term (over 24 weeks). Abbreviation: PRP, platelet-rich plasma.

**Figure 4:**
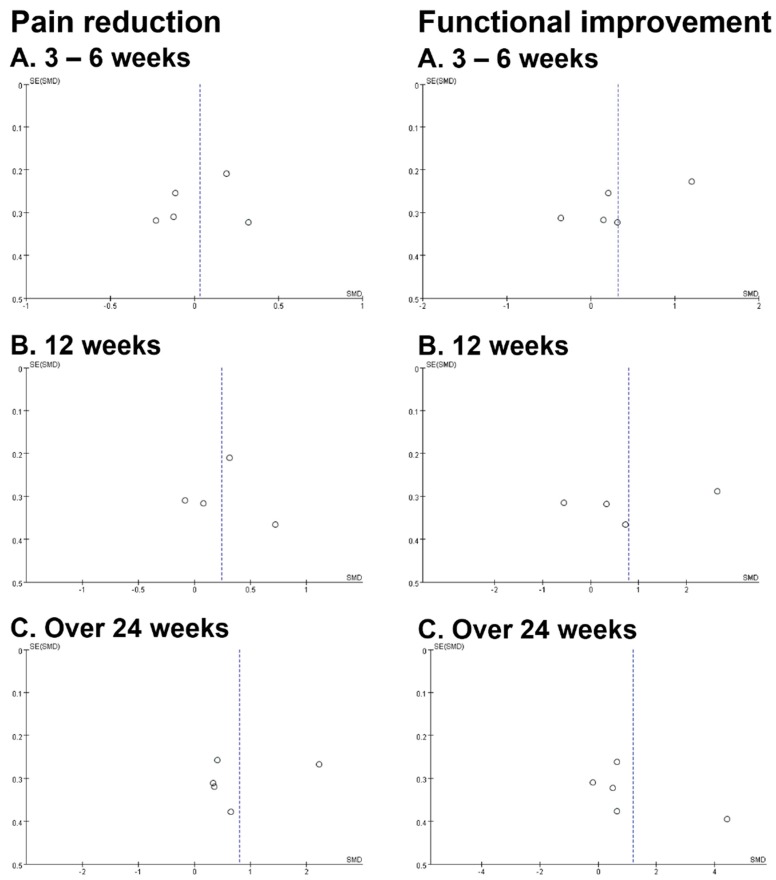
Funnel plots showing publication bias for pain reduction (left) and functional improvement (right) at the short term (**A**, 3–6 weeks), the medium term (**B**, 12 weeks), and the long term (**C**, over 24 weeks).

**Figure 5:**
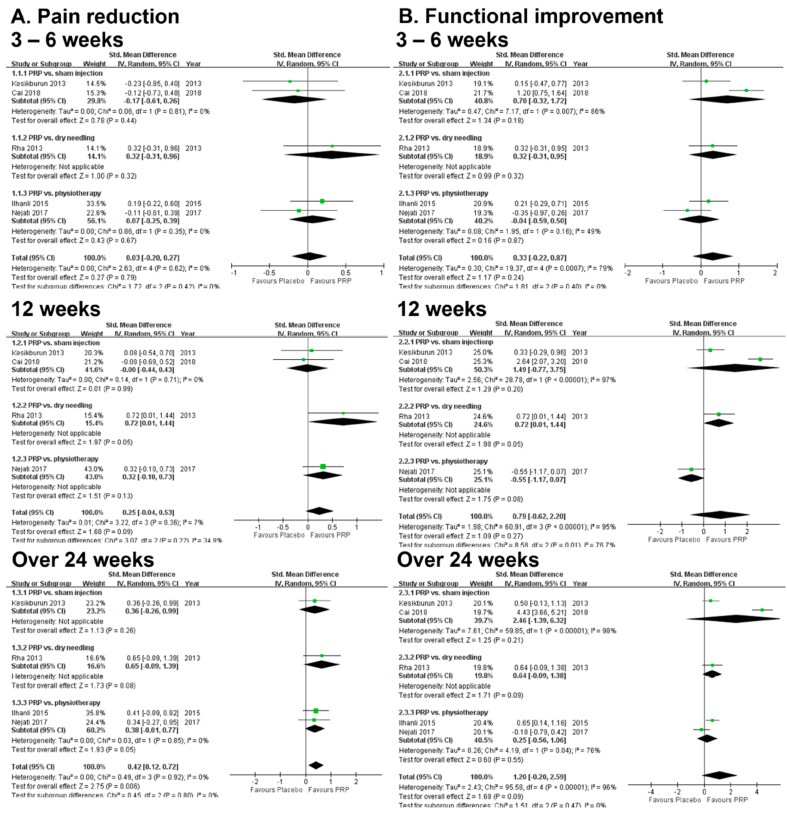
Forest plots of subgroup analysis: comparison between PRP and control groups including sham injection, dry needing, and physiotherapy in pain reduction (**A**) and functional improvement (**B**) at the short term (3–6 weeks), the medium term(12 weeks), and the long term(over 24 weeks). Abbreviation: PRP, platelet-rich plasma.

The forest plot of pooled SMDs to compare the outcome of pain reduction between the PRP and control groups is presented in Figure 3. In the short and medium terms, the difference between the PRP and control groups was not significant (Supplementary Figure S1). The PRP group reduced pain more effectively only in the long term but with significant heterogeneity when all five studies were included for analysis (SMD: 0.81, 95% CI: 0.03–1.58, I^2^ = 0.88, Supplementary Figure S1). Therefore, a sensitivity analysis was conducted for high heterogeneity after one low-quality study was excluded [14]. The result of the sensitivity analysis still revealed a significant long-term benefit of PRP in pain reduction (SMD: 0.42, 95% CI: 0.12–0.72) and the heterogeneity was eliminated (I^2^ = 0) (Figure 3). Publication bias was not found based on the symmetry of the funnel plot (Figure 4). In the subgroup analysis stratified by three different control groups (sham injection, dry needling, or physiotherapy), the difference between the PRP and each subgroup group was not significant (Figure 5) for pain reduction in the short, medium, and long terms. 

### 3.4. Results of Meta-Analysis: Secondary Outcome (Functional Improvement)

The PRP group had a slightly better functional outcome, but the effect was not significant in the short term (SMD: 0.33, 95% CI: −0.22–0.87), medium term (SMD: 0.79, 95% CI: −0.62–2.20), or long term (SMD: 1.20, 95% CI: −0.20–2.59) (Figure 3). Publication bias was not found based on the symmetry of the funnel plot (Figure 4). In the subgroup analysis stratified by three different control groups (sham injection, dry needling, or physiotherapy), the difference between the PRP and each subgroup group was not significant (Figure 5) for functional recovery in the short, medium, and long terms.

### 3.5. Meta-Regression

There was no association between number of injections and pain reduction, and neither was there association between number of injections and functional recovery (all *p* values > 0.05).

### 3.6. Adverse Effect

There was no report of marked complications in the PRP or control group [10,11,12]; no information on therapeutic complications was available in two of the included RCTs [13,14]. The only documented complication was pain that lasted for few days after PRP injection [10,11,12]. 

## 4. Discussion

Our meta-analysis demonstrated that PRP injection provided probable benefit in pain reduction in the long term over control group including sham injection, no injection, or physical therapy alone among patients with rotator cuff tendinopathy. As for the functional outcome, there was no significant difference between the PRP group and the control group.

PRP injection has been used widely in regenerative medicine. Tendinopathy is a highly prevalent tendon disorder and plagues a range of individuals from common persons to elite athletes; however, its underlying mechanisms are not fully understood. The model of degeneration or overuse injury has been wildly accepted to explain the pathophysiology of tendinopathy in the past decade [23]. It has been hypothesized that, instead of inflammation, the main cause of chronic tendinopathy is the insufficient healing potential [24]. Based on these concepts, regenerative medicine was developed to promote tissue healing. Platelets are known to release growth factors, cytokines, and chemokines to modulate inflammation and tissue regeneration [9]. Previous in vitro studies have shown that PRP, prepared by centrifugation to increase the concentration of platelets and growth factors, can promote the proliferation of tenocytes and facilitate tendon repair [25]. With the present meta-analysis, we found that the currently available clinical evidence on PRP injection supports a beneficial effect on pain reduction in rotator cuff tendinopathy.

The effect of injections using different autologous blood-derived products on treating various musculoskeletal disorders including knee osteoarthritis, hip osteoarthritis, tennis elbow, and rotator cuff has been studied in the past decades [8,9,15]. Nonetheless, evidence remains uncertain of the effectiveness of PRP as an adjunct used in rotator cuff repair surgery [26]. Recently published reviews have shown benefits of PRP in rotator cuff tendinopathy in the long term [15,27]. The analysis conducted by Chen et al. showed that patients treated with PRP for rotator cuff injuries and lateral epicondylitis reported significantly less pain [27]. There were three more recently published studies included in this meta-analysis [12,13,14]. The current meta-analysis showed that PRP is effective in long-term pain reduction among patients with rotator cuff tendinopathy, and the result is in agreement with existing literature. The superiority of PRP over the control group including physical therapy, sham injection with saline, or dry needling may be due to increased regenerate tissue homeostasis and stronger therapeutic effects with PRP injection [10,12]. 

In this meta-analysis, no significant functional benefit in the long term was observed for PRP treatment in comparison with the control group. A prior study disclosed significant functional benefits in the long term with PRP injection, but the article did not consider patients who were treated with exercise therapy [15]. Exercise therapy has been regarded as one of the standard treatments for rotator cuff tendinopathy with its possible beneficial effect in tendon homeostasis, preventing negative effects of immobilization and aiding collagen turnover [28]. A previous study reported a significantly better functional outcome, sustained for 6 months with exercise therapy compared with placebo in rotator cuff disease [16]. In addition, the benefit of exercise therapy including supervised exercise and home exercise program over placebo or no intervention in both pain and functional outcome have been supported by the review done by Little et al. [3]. Our study was the first one to investigate the effectiveness of PRP injection over a control group comprising not only placebo and sham injection but also exercise therapy and physical therapy. Nonetheless, only two RCTs comparing PRP injection with exercise therapy or physiotherapy were included in the current study [12,13], and the results of subgroup analysis did not reveal a positive outcome for pain reduction or functional recovery (Figure 5). Nevertheless, whether PRP injection could provide further functional benefits over exercise therapy (or physiotherapy) requires further investigation.

Injection therapies with PRP are considered safe based on existing evidence [29]. Rare and predominantly minor complications have been reported following PRP use [29]. Complications including swelling, tenderness, joint pressure, and local pain are associated with the distension of the joint caused by intra-articular injection [30]. As for intralesional injection for treating tendons and ligaments, local pain at the injection site is the main complaint [29]. In our study, no data of adverse effects were reported in two of the included studies [13,14] and only pain lasting for a few days after injection was documented in others [10,11,12]. Intra-articular injection was conducted in one of the included studies in which only pain was reported [12]. Based on the results of previously published literature and our current analysis, intralesional or intra-articular PRP injection is a safe and well-tolerated treatment for rotator cuff tendinopathy.

## 5. Limitations

There were several limitations in this meta-analysis. First, there was heterogeneity in the diagnostic criteria among different trials. The patient groups included in different studies also varied. Rotator cuff pathology including supraspinatus tear, supraspinatus tendinosis, rotator cuff tear, rotator cuff tendinosis, and subacromial impingement was eligible for enrollment in different studies. In addition, image confirmations via MRI or ultrasonography were utilized for diagnosis. Thus, it was not possible to categorize these RCTs accurately for a subgroup analysis stratified by clinically diagnosed and image-diagnosed subgroups. Second, the guidance technique for injection either to the subacromial space or injured tendon may influence the treatment effect of PRP. Four of the included studies in our meta-analysis used ultrasound guidance, and no descriptions regarding guidance method were available in the remaining study. Therefore, we could not compare the potential effect of different guidance methods. Third, the interventions in the control group varied, including dry needling therapy, saline injection, and physical therapy. Fourth, no detailed information about the composition of PRP including platelet concentration, leukocyte concentration, biochemical analysis, and preparation method was available. Therefore, the development of therapeutic guidance, high-quality studies with well-documented PRP compositions, and standardized classification systems are warranted. Additionally, cointervention with self-exercise or home program were conducted in three of the enrolled studies [11,12,13]. Although multiple treatment modalities might be needed to reach optimal treatment outcome in treating rotator cuff tendinopathy considering the complexity of the disease entity [7], the impact of the cointervention on PRP injection could not be evaluated.

It is possible that PRP injection could provide a more beneficial effect in treating rotator cuff tendinopathy according to the result from our study. Nonetheless, heterogeneities including different preparation and technique used in PRP injection, various treatment options in the control group in the enrolled studies, a small number of studies and co-interventions used were factors that may have influence on the results. To confirm the positive effects implied by this meta-analysis, more double-blinded randomized controlled trials with a larger study population are required to be analyzed in the future.

## 6. Conclusions

The present meta-analysis indicated that PRP injection may be more effective than the control group (sham injection, no injection, or physiotherapy alone) in reducing pain in the long term (over 24 weeks) for patients with rotator cuff tendinopathy. PRP injection did not provide significant benefit in functional improvement. Additionally, subgroup analysis comprising PRP and physiotherapy showed no significant between-group difference considering pain reduction and functional outcome in this study. Further investigation is needed to disclose whether PRP injection could provide benefit over physiotherapy.

## Figures and Tables

**Table 1:** Summary: the characteristics of included studies.

Reference	Study/LOE	Interventions	Inclusion Criteria	Number	Age	Symptom Duration	Injection/Interval	Rx dose/Guidance Method and Injection Location	Co-Interventions	Outcome Measure	Follow-Up Weeks	Adverse Effect
Rha et al., 2013 [10]	RCT/ level 1	PRP vs. dry needling	Supraspinatus tendon lesion (tendinosis or a partial tear)	20/19	52.2/53.9	9.6/9.2 M	2/4W	PRP: 3 mL of PRP(Prosys^®^) extracted from centrifuged 25 mL of patients’ blood Placebo: dry needling / Method: ultrasound guided; supraspinatous tendon	Self-exercise rehabilitation program	SPADI, ROM	2W, 4W, 6W,12W, 24W	Pain lasting for few days
Kesikburun et al., 2013 [11]	RCT/ level 1	PRP vs. sham injection	Rotator cuff tendinosis or partial tear diagnosed by MRI	20/20	45.5/51.4	8.5/10 M	1/NA	PRP: 5 mL of PRP (GPS III Platelet Separation System) extracted from centrifuged 54 mL of venous blood Placebo: 5 mL of saline / Method: ultrasound guided; subacromial	Exercise program (supervised by PT), then home program	VAS, SPADI, WORC, ROM	3W, 6W, 12W, 24W, 1Y	Local pain lasting for few days
Ilhanli et al. 2015 [12]	RCT/ level 1	PRP vs. physiotherapy	Supraspinatus partial tear diagnosed with MRI	35/35	59.2/59.7	7.3/7.2 M	3/1W	PRP: 6 mL of PRP extracted from 15 mL of peripheral blood PT: hot packing, ultrasound, TENS, ROM exercise, stretching and strengthening exercise /Method: Intra-articular injection	PT program after end of injections	ROM, VAS, DASH, BECK	End of the treatment, 12M	Pain
Nejati et al. 2017 [13]	RCT/ level 1	PRP vs. physiotherapy	Subacromial impingement syndrome via clinical assessment and MRI	22/20	52.5/53.9	Not reported	2/1M	PRP: 4 mL of PRP (Tubex Autotube System) extracted from centrifuged 25 mL of venous blood Exercise therapy: supervised and self ROM exercise and strengthening exercise /Method: ultrasound guided; torn or injured tendon and subacromial space	Nil	VAS, ROM, DASH, WORC, MRI findings	1M, 3M, 6M	Not reported
Cai et al. 2018 [14]	RCT/ level 1	PRP vs. sham injection	Partial-thickness rotator cuff tears diagnosed clinically and via MRI	45/47	40.6/39.9	14.1/13.5W	4/1W	PRP: 4 mL of PRP extracted from centrifuged 20 mL of autologous venous blood Placebo: 4 mL of saline /Method: ultrasound guided; subacromial injection	Nil	ASES, Constant score, VAS, AP tear size on MRI	1M, 3M, 6M, 12M	Not reported

BECK Beck Depression Inventory Score; LOE, Level of evidence; RCT, Randomized controlled trial; RC, rotator cuff; Rx, treatment; PRP, platelet-rich plasma; HA, hyaluronic acid, PT, physiotherapy; NSAID, nonsteroidal anti-inflammatory drugs; VAS, Visual Analogue Scale; NRS, Numeric Rating Scale; ASES, American Shoulder and Elbow Surgeons Shoulder Score; SDQ, Shoulder Disability Questionnaire; DASH, Disabilities of the Arm, Shoulder and Hand Score; Oxford Shoulder Score, OSS; WORC, Western Ontario Rotator Cuff Index; SPADI, Shoulder Pain and Disability Index; SST, Simple Shoulder Test; TENS, Transcutaneous Electrical Nerve Stimulation; USPRS, Ultrasound Shoulder Pathology Rating Scale; ROM, range of motion; W, week(s), M, month(s), Y, year(s).

## Supplementary Materials

The following are available online at https://www.mdpi.com/2075-4418/10/4/189/s1, Supplementary-Methods: s-Methods; Figure S1: Forest plots of meta-analysis: comparison between PRP injection and placebo in pain reduction (left) and functional improvement (right) at short term (A, 3–6 weeks), medium term (B, 12 weeks), and long term (C, over 24 weeks). Abbreviation: PRP, plate-rich plasma.

## Abbreviations

PRP	Platelet-rich plasma
RCT	Randomized controlled trial
SD	Standard deviation
SMD	Standardized mean difference
CI	Confidence interval
