# Supplementary material for: Effectiveness of Platelet-Rich Plasma Injection in Rotator Cuff Tendinopathy: A Systematic Review and Meta-Analysis of Randomized Controlled Trials
Source: Diagnostics (Basel). 2020 Mar 28;10(4):189. doi: 10.3390/diagnostics10040189 (PMC7235747; doi:10.3390/diagnostics10040189)
Supplement: Supplementary file 1 [file diagnostics-10-00189-s001.pdf]

s-Methods

Identification of trials and search method

Text words with key terms for electronic databases:

1. PubMed, The Cochrane Collaboration Central Register of Controlled Clinical Trials and Cochrane Database of Systematic Reviews :

(“rotator cuff" OR subacromial OR sub-acromial OR impinge* OR supraspinat* OR infraspinat* OR subscapular* OR “teres minor”)

AND (autologous OR platelet or plasma OR PRP OR platelet rich plasma OR platelet gel OR platelet derived growth factors OR platelet concentrate OR PRGF OR ACP OR autologous conditioned plasma OR platelet lysate OR platelet rich fibrin OR platelet rich membrane)

2.EMBASE and Scopus:

#1 'rotator cuff'/exp OR 'rotator cuff’

#2 'subacromial'/exp OR impinge* OR supraspinat* OR infraspinat* OR subscapular* OR ‘teres minor’

#3 #1 OR #2

#4 ‘autologous’ OR ‘platelet’ or ‘plasma’ OR ‘PRP’ OR ‘platelet rich plasma’ OR ‘platelet gel’ OR ‘platelet derived growth factors’ OR ‘platelet concentrate’ OR ‘PRGF’ OR ‘ACP’

#5 arthroscop* OR arthroplas* OR acromioplas* OR ‘adhesive capsulitis’/exp OR ‘rat’/exp OR ‘mice’/exp OR ‘animal’/exp)

#6 #3 AND #4 NOT #5

Data synthesis and analysis

Pooled SD $_{intervention}$= the square root of {[(participant numbers $_{baseline}$ – 1)*(SD $_{baseline}$)^2 + (participant numbers $_{post\text{-}intervention}$ – 1)*(SD $_{post\text{-}intervention}$)^2] / [(participant numbers $_{baseline}$ – 1) + (participant numbers $_{post\text{-}intervention}$ – 1)]}.

Pooled SD $_{between\text{-}interventions}$= the square root of {[(participant numbers $_{First\ intervention}$ – 1)*(pooled SD $_{First\ intervention}$)^2 + (participant numbers $_{Second\ intervention}$ – 1)*(pooled SD $_{Second\ intervention}$)^2] / [(participant numbers $_{First\ intervention}$ – 1) + (participant numbers $_{Second\ intervention}$ – 1)]}.

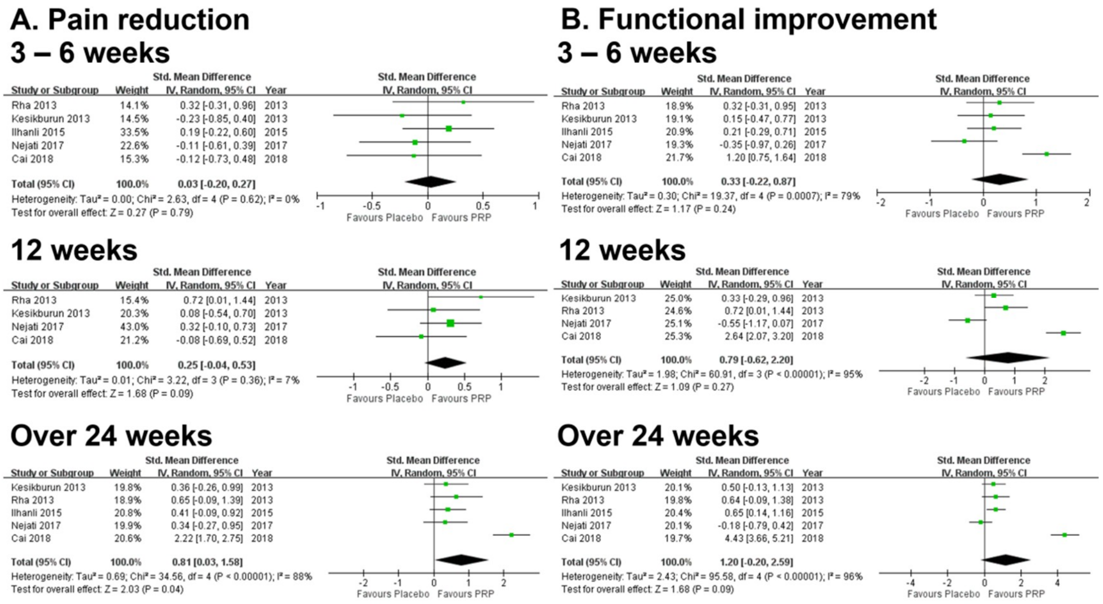

Supplemental Fig S1. Forest plots of meta-analysis: comparison between PRP injection and placebo in pain reduction (**left**) and functional improvement (**right**) at short term (A, 3–6 weeks), medium term (B, 12 weeks) and long term (C, over 24 weeks). Abbreviation: PRP, plate-rich plasma.
